# An ICT-Based Diabetes Management System Tested for Health Care Delivery in the African Context

**DOI:** 10.1155/2014/437307

**Published:** 2014-07-17

**Authors:** Claude Takenga, Rolf-Dietrich Berndt, Olivier Musongya, Joël Kitero, Remi Katoke, Kakule Molo, Basile Kazingufu, Malikwisha Meni, Mambo Vikandy, Henri Takenga

**Affiliations:** ^1^Infokom GmbH, 17034 Neubrandenburg, Germany; ^2^Health Department, Baptist Church in Central Africa (CBCA), Goma, Democratic Republic of Congo; ^3^Ruwenzori State University (UOR), Butembo, Democratic Republic of Congo; ^4^Provincial Health Division (DPS), Ministry of Health, North Kivu, Goma, Democratic Republic of Congo

## Abstract

The demand for new healthcare services is growing rapidly. Improving accessibility of the African population to diabetes care seems to be a big challenge in most countries where the number of care centers and medical staff is reduced. Information and communication technologies (ICT) have great potential to address some of these challenges faced by several countries in providing accessible, cost-effective, and high-quality health care services. This paper presents the Mobil Diab system which is a telemedical approach proposed for the management of long-term diseases. The system applies modern mobile and web technologies which overcome geographical barriers, and increase access to health care services. The idea of the system is to involve patients in the therapy process and motivate them for an active participation. For validation of the system in African context, a trial was conducted in the Democratic Republic of Congo. 40 Subjects with diabetes divided randomly into control and intervention groups were included in the test. Results show that Mobil Diab is suitable for African countries and presents a number of benefits for the population and public health care system. It improves clinical management and delivery of diabetes care services by enhancing access, quality, motivation, reassurance, efficiency, and cost-effectiveness.

## 1. Introduction

The number of people with diabetes is increasing due to population growth, aging, urbanization, and increasing prevalence of obesity and physical inactivity [1]. Diabetes is associated with huge human and economic costs. Predictions show that the number of people with diabetes worldwide will double in the next generation. In [2] the quality of diabetes management in five countries with advanced economies (Australia, Canada, New Zealand, USA, and UK) has been analysed. Yearly costs (e.g., 132 Billions USD in the USA) and deaths due to diabetes (e.g., 143 308 people in the USA) are reported in that paper. In face of such a disastrous situation, nations have intensified their efforts to struggle with diabetes.

Improving accessibility of the African population to diabetes care seems to be a big challenge in most of African countries such as the Democratic Republic of Congo (DRC), a country which is four times bigger than France and where the number of care centers is reduced. DRC has 71 million inhabitants. Around 7% of the population in the capital city Kinshasa is affected by diabetes [3]. This situation is similar in other African countries. Most of diabetic patients in the DRC do not control regularly their glucose values, occasional checks are the only executed options.

What are the factors which are responsible for deficiency in the successful management of diabetes in Africa? A number of obstacles make the detection, prevention, monitoring, and treatment of diabetes difficult in most African countries [4].

The first group of difficulties is related to the health facilities which are not sufficient to cope with the scale of the problem. Prevention and education are not sufficiently promoted, although they should be given high priority. There are not enough medical specialists and the few existing focus their work in big cities without addressing the needs of rural populations. Therefore, patients from rural areas are forced to spend money and time travelling long and expensive journeys to access diabetes care. Another point, the priority of health politics in several African countries is oriented on the management of transmissible diseases such as HIV and not to chronic diseases like diabetes and high blood pressure.

The second group of difficulties is related to patients themselves. It is impossible for the majority of the population to afford the cost of the diabetes medication in particular insulin. Due to life condition, patients are unable to control a proper diet, especially when the family eats by hand in the same dish. Limited resources push the population to focus on high calorie foods rich in fat, sugar, and salt.

The third group of difficulties is related to the sociocultural context which has a considerable weight in Africa. Africans have difficulty accepting the concept of chronic illnesses requiring lifelong treatment, so when diabetes is well balanced, patients often stop the treatment thinking the disease is cured. This then causes a relapse in the following days. Moreover, patients do not find it useful to carry out regular glucose checks although this is necessary for the adaptation of the insulin therapy. The next point deals with the fact that the struggle against obesity faces the belief that being fat is a sign of wealth, good health, and beauty. According to the African tradition, women beauty is proportional to weight. Weight loss, even if it is wanted, raises suspicion of possible HIV infection in the continent. Some religions create obstacles to the diabetes management in Africa. Fasting in churches and during Ramadan is another source of poor glucose control. The last obstacle is caused by traditional medicine which plays a key role in many African countries. This practice is often dangerous when it advices diabetes patients to stop effective treatments and use empirical drugs which have the advantage of being cheap.

Information and communication technologies have great potential to address some of the challenges faced by the public health care. A number of literature have been oriented on the exploitation of mobile devices for completing traditional therapy [5, 6]; other works have been focused on the architectural issues of a telemedicine intervention [7, 8].

A significant number of telemedical products in the area of diabetes management have been developed in the last decade. This is justified by the fact that recent studies have depicted a very critical situation; over 10% of the United States population over the age of 20 has diabetes. This number has been predicted to increase significantly in the future. As a result, diabetes is now ranked amongst the ten first leading causes of death. This situation motivates special attention towards both prevention and better diabetes treatment.

A web-based system for monitoring patients with type 2 diabetes is presented in [9]. In that system weight, blood sugar, and blood pressure are recorded using an application implemented on the mobile phone. In [10] Bayer's GLUCKOFACT DELUXE Software is described. The software recognizes the Bayer glucose meter connected to the personal computer and stores automatically the data in a database. The visualization of the recorded data is well organized. However, that software is not extended for online applications. This obliges the patient to regularly visit his doctor or to use another system in order to send the recorded data. A more advanced diabetic management system is presented in [11]. The software helps in tracking daily diabetic data; it has a simple interface for data entry and uses different devices for entering data (Windows-PC, Android phones, Windows mobile phones, iPhones, and Java phones). It has been proved in [12, 13] that telemedicine is a suited instrument to support health care providers in the effective management and prevention of diabetes complications.

Solution to the four listed main difficulties in properly addressing diabetes in Africa can be solved by providing a cost-effective telemedical system and a suitable educational program in order to increase the population awareness. This is the focus of this paper, in which we propose and validate an innovative mobile health application. The system manages a secure collection, processing, storage, and sharing of diabetes-related data and thus reduces the frequency of physical consultations to the treating physician. The system enables the organization of health information through a structured gathering of all relevant diabetes data in one central place enabling only access of authorized medical staff. The system helps improve the quality of service in the care process of diabetes by benefiting from rapid advances in mobile, web, and communication technologies. The system also provides educational programs in order to increase awareness on diabetes. We strongly believe that the outcome of this work will contribute toward the establishment of a model for diabetes management in countries where access to diabetes care is limited and number of medical care personnel is reduced due to high personnel costs. The diabetes management system Mobil Diab, presented in this paper, can serve as a complete hospital management system which supports several hospitals, each hospital having its own medical staff and patients and every clinician coaching a group of patients assigned to him. Moreover, since e-health systems store sensitive data and should have a proper security and privacy framework and mechanisms, this paper extends the state-of-the art by presenting a system which enables a secure data access, communication, and storage. Investigation of the impact of using this system Mobil Diab in assisting the therapy and treatment of diabetes is the major focus of this paper.

## 2. Materials and Methods

### 2.1. Mobil Diab System and Benefits

Mobil Diab system is an innovative solution for the assistance and care of diabetes patients. It enables diabetes patients to manage their self-control data around the clock using their mobile devices (Android, iPhone, and iPads) and/or web-based applications. Medical care personnel access patients' data through a protected web portal. The diabetes module is embedded on the telemedical platform. The platform is responsible for the following tasks: connection of web and mobile applications, user-hierarchical model (administration, hospital, doctor, and patient), access control of different user categories, and secure interface to hospital information systems.

**Figure 1:**
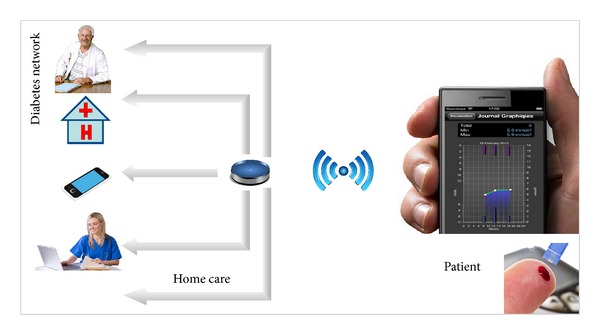
Mobil Diab system components.

The concept of the system aims at empowering patients to better manage their disease by allowing them to monitor their blood sugar trends over an extended period of time and draw some actionable conclusions. Acquired data are then sent using HTTP/HTTPS protocol to the central platform which ensures a secure storage and further processing of the information, Figure 1.

Mobil Diab guarantees a series of benefits for patients, health care staff, and public health system.

Benefits for the patients include, among others, unimpeded patient mobility, data input via smart phones and/or via web, regular self-control of diabetes-related data enables the right care to be administrated at the right time, potential to improve care process and quality of service, improvement in patient's motivation through their involvement in the therapy process, reduced check-up frequency to doctors, and use of mobile health technologies encourages diabetes patients to change their behavior/lifestyle and improve their health.

Benefits for the health care staff involve, among others, the following: complete and regular data input which is helpful for individual therapy plan, minimization of errors caused by lack of information about the disease history, improvement of the care process quality, getting specialist opinions, access to patients' data worldwide independent from time and location, and automatic alarm message in case of critical data from a patient.

Benefits for the Health System include, among others, the following: delaying and reducing diabetes complications, minimizing hospitalization rates due to diabetes complications, reducing death rates from diabetes, speeding up the transition of patients from hospitals to their own homes which leads to a reduction in costs, and enabling the organization of health information through a structured gathering of all relevant data in one central place.

The system helps to easy track patterns and trends in diabetes management process, helps in taking right treatment decisions and lead to a better glucose control, is suitable for self-care, home care, family physicians, clinics, and hospitals, presents statistics that cover everything doctors and patients need, allows risk monitoring with automated alarm message to care provider and trusted person, preserves mobility through the use of Apps and web technologies, and acquires a highly secured database platform with end-to end encryption and data are worldwide available in all major languages.

### 2.2. Mobile Application of Mobil Diab

**Figure 2:**
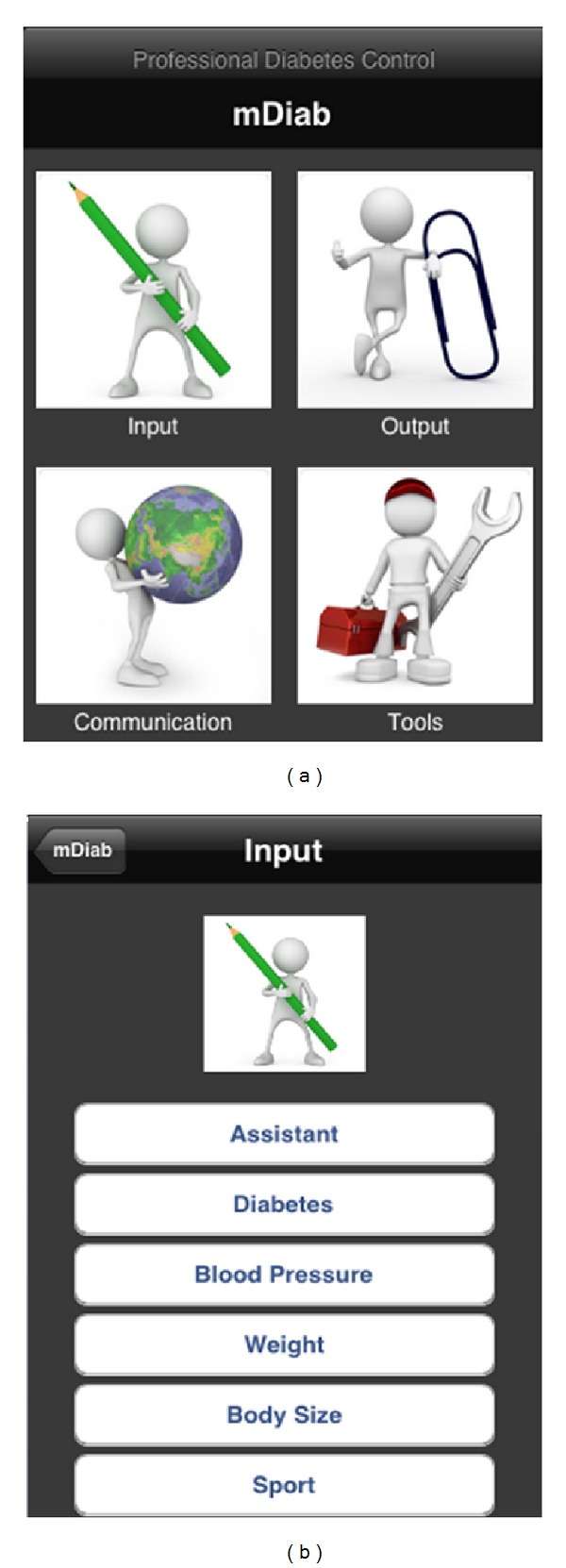
Mobil Diab mobile application: screenshots of the main screen and the input choices.

**Figure 3:**
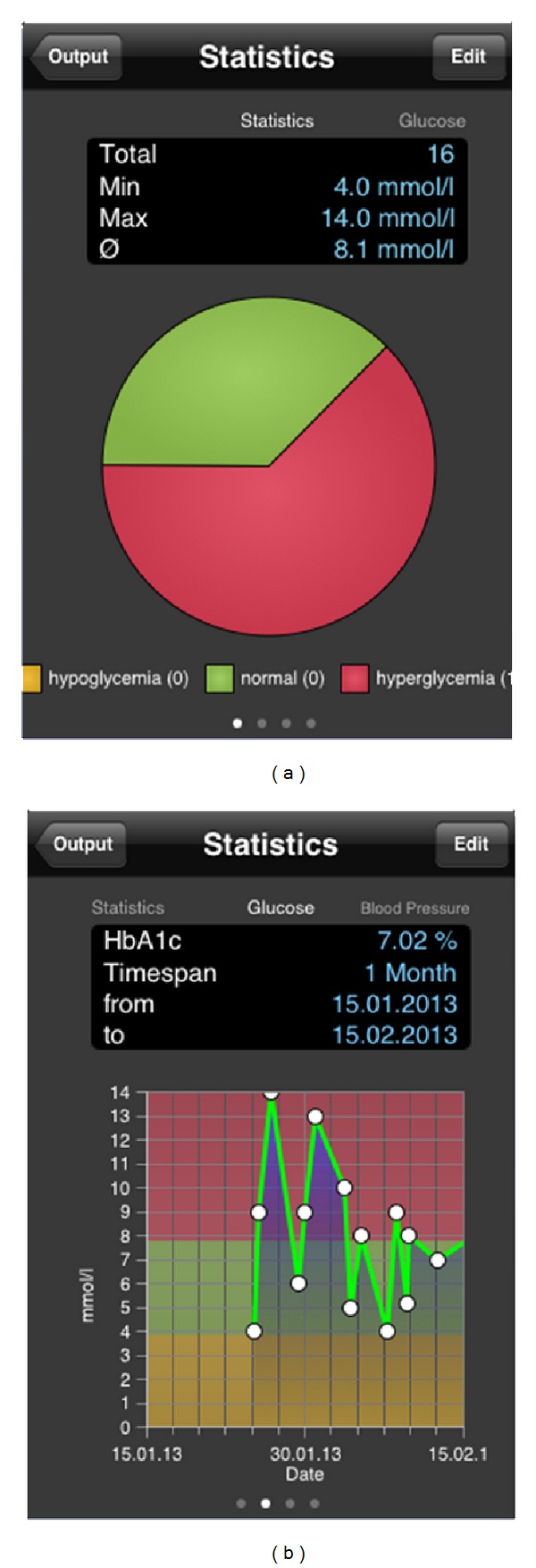
Mobil Diab mobile application: output screens and blood glucose graph.

The mobile application Mobil Diab has an intuitive user interface which is self-explained. You can choose the action you wish to perform as shown on the screenshot of Figure 2. Results are also represented in different ways as samples are shown in Figure 3. Data are automatically synchronized in background with the online system so that medical care providers receive these data in real time.

To record diabetes-related data, patients are presented with intuitive screens and they can enter data. These data may include, amongst others, the blood glucose measurement, insulin intake, sport done with duration, blood pressure measurements, and body weight and size. Data acquisition can also take place automatically from glucose meters supporting the IEEE 11073 Personal Health Data Standards. Data synchronization is done automatically and in two directions.

Therapy plans, instructions, and recommendations sent by the doctor from the doctor portal are received directly in the mobile application. For patients without smartphones (Mobil Diab app), they can still get these doctors' feedbacks in the protected patients' portal, through their email address or as sms if this service was activated.

### 2.3. Web-Based Applications of Mobil Diab

The web structure of the system is conceived in order to serve as a complete hospital management system. Four web-based portals are integrated to the system and hosted from the central platform. They are designed for four different types of users: patients, doctors, hospital administrators, and system administrator.

**Figure 4:**
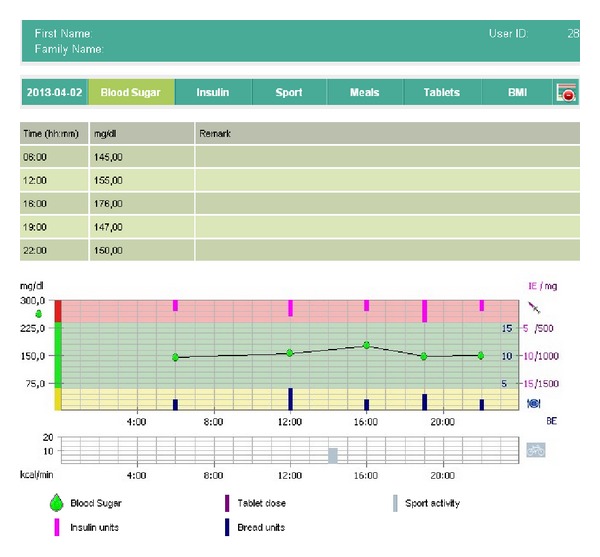
Mobil Diab web application: doctors' portal and glucose day graph.

Figure 4 is a section of the screenshot of the doctor portal. The graphic shows the glucose measurements of one day, amount of carbohydrate taken (blue color facing up), and units of insulin injected (purple color facing down). Clinicians have a structured and understandable view of patients' data. Clear graphical representation of trends and statistics enables clinicians to make appropriate decisions on therapy adjustment. The tool helps medical staff save their times while providing high quality service to diabetes patients.

Doctors can receive messages from their patients directly in this portal. For emergency cases, an SMS is automatically generated and sent to the treating physician, to alert him to check the critical situation in the portal and be able to give direct instructions to the patient.

### 2.4. Architecture of the Telemedical Platform

**Figure 5:**
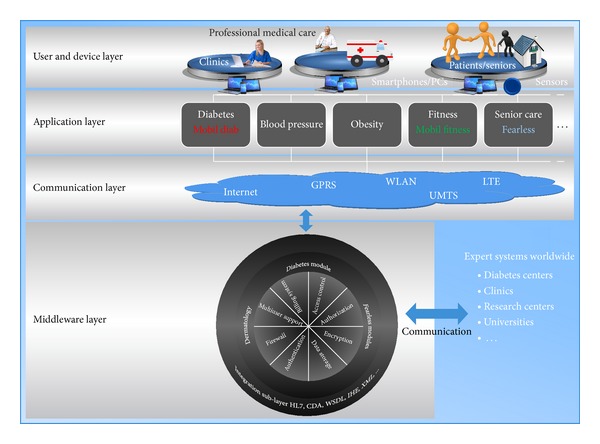
Architecture of the telemedical Platform.

The patent-protected platform illustrated by Figure 5 has been developed to help bridge the gap between health and mobility. Diverse healthcare modules have been implemented and other are planned in order to meet a complete health care service package from diabetes, dermatology, stress, fitness, and long-term health conditions up to the assisted-living for senior. The platform helps consumers track health, wellness, and vital information using a highly secure infrastructure. It allows consumers to share information with their health care professionals and family. Mobility is guaranteed by integrating mobile Apps and web-based applications. Moreover, interfaces to hospital information systems and practice management software are supported. In this work, the diabetes module was further developed, updated, tested, and validated. The four-layer architecture of the platform enables users to securely share sensitive information. Using different devices, such as smartphones and computers, users can access functionalities of applications supported by the core of the platform through the communication layer.

Security is a general requirement in modern computing environments, but in e-health systems security is an imperative requirement because those systems handle very sensitive data like medical and personal data. The platform acquires features enabling the following.

- Authentication: methods and mechanisms which allow an entity to prove its identity to a remote end.
- Authorization: access control mechanisms and the ability of an entity to access shared resources.
- Data integrity: mechanisms which ensure that when there is an interchange of data between two peer entities, the received data and the original ones are the same, and that no intermediate alteration has occurred.
- Data confidentiality: it assures that stored or transmitted data are well protected from possible disclosure. A mean used to achieve data confidentiality is through cryptographic mechanisms.
- Privacy: it can be defined as an entity's ability to control how, when, and to what extent personal information about the entity will be communicated to third parties.
- Secure data communication and storage.
- Data availability: data can be accessed by authorized users independently from time and location.

Our proposed architecture addresses authentication and authorization issues, since each user is classified to a category that defines what he/she has access to. Moreover, data confidentiality is achieved through cryptographic mechanisms. Medical data and personal data are encoded and stored in separate data bases. All data are encrypted using the symmetric encryption method AES (Advanced Encryption Standard). For the protection of user's privacy, an implemented mechanism allows different groups of users to have access to different types of medical data.

The platform is composed of several modules, each one supporting its group of tasks. This structure makes the support of new services easier. The integration sublayer is responsible for formatting data in a needed standard. This extends the interoperability capability with other third-party systems. Some of the supported standards include HL7, CDA, WSDL, and XML. This platform was also presented in [14–16].

### 2.5. Experimental Settings and Recruitment Criteria

In order to test the effectiveness of the diabetes management system Mobil Diab in the context of African health care system, a trial was conducted in two cities in the eastern part of the Democratic Republic of Congo (Goma and Butembo). Patients diagnosed with type 2 diabetes and aged between 35 and 75 years were recruited randomly. A total of 40 patients were included in the trial phase. For classification and evaluation purpose, the cohort was divided into a control group (conventional therapy without the use of telemedicine system) and an intervention group (treatment with the use of telemedicine system Mobil Diab). To verify the comparability of the two groups, Table 3 shows that the control and intervention groups in terms of anthropometric data and metabolic control parameter are almost the same at the beginning of the trial: the mean age for the intervention group is 53.3 years with a standard deviation of 10.7 years and for the control group is 53.35 years with a standard deviation of 9.59 years. The mean HbA1c value for the whole intervention group before the trial was 8.67% and for the control group was 8.59%. The trial was conducted for a period of two months. During this trial period, medical staff of four local hospitals belonging to the health department of the Baptist Community in Central Africa (CBCA) accessed the system and coached the patients based on the analysis of the sent data.

At the end of the trial each participant had to fill a questionnaire evaluating the system Mobil Diab based on the three following metrics: usability and design, efficiency and therapy satisfaction, and acceptance and appreciations. Questions for patients were as follows.

- Q1: How often could you successfully use the system?
- Q2: How easy was it for you to cope with the application?
- Q3: How do you evaluate the input options and design of the application?
- Q4: How do you evaluate the output options and visualization possibilities?
- Q5: How do you evaluate the design of the application?
- Q6: Do you find Mobil Diab as a tool which motivates you in the control of your blood glucose levels?
- Q7: Did feedbacks (messages and therapy) from doctors help in the diabetes management process?
- Q11: Would you wish to continue using Mobil Diab for managing your diabetes?
- Q12: Would you recommend the system to other users?

Each medical care personnel had to evaluate the system by answering the following questions.

- Q1: How difficult was it for you to use the doctor web application to interact with your patients?
- Q2: How do you evaluate the design of the doctor portal?
- Q3: How often could you successfully use the system?
- Q4: How do you evaluate the representation of diabetes data in the portal?
- Q5: How do you evaluate the monthly representation of patient's data on graph?
- Q6: How do you evaluate the feature for report generation?
- Q7: How do you appreciate the page summarizing all important patient's data at glance?
- Q8: Is the provided information in portal enough for improving the therapy adaptation?
- Q12: Would you wish to continue using Mobil Diab for coaching your diabetes patients?
- Q13: Would you recommend the system to other medical care personnel?

A 10-point scale from 1 to 10 was used for questionnaire, with higher scores representing a better evaluation of the system based on the given question. Patients and medical care staff provided their evaluations as presented in Tables 1 and 2. Moreover, based on the analysis of patients' data collected during the trial period, the improvements in clinical outcome could be evaluated. Two metrics were used for this purpose: the mean amplitude of glycemic excursions (characterized by the mean blood glucose and its standard deviation) and the glycated hemoglobin (HbA1c), as Table 3 presents the results.

**Figure 6:**
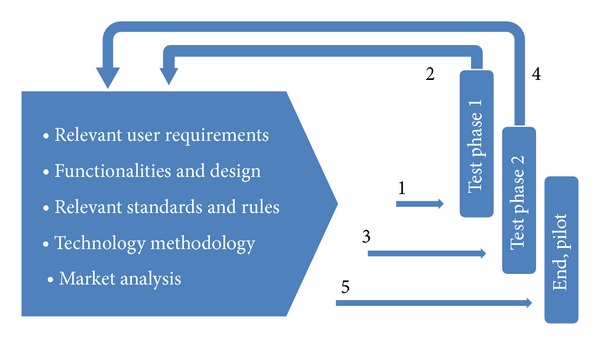
Flow of the trial process.

Before the trial could start, some settings, adaptations, and tests were made. All the involved partners (patients, medical care providers, support teams, administrators, internet and telephone providers, developers, and policy makers) worked closely together during the development and adaptation process of the system.  Figure 6 illustrates the flow of the trial. For each test stage, user requirements, functionalities and design, relevant standards and rules, and methodology and market analysis were discussed with all the involved players. Analysis of feedbacks from each stage constituted important input for the following stage. At the end, final modifications were made to obtain the final pilot ready for use in a larger scale.

Through collaboration with the local university, the Ruwenzori State University in Butembo, Democratic Republic of Congo, both its faculty of medicine and faculty of applied sciences will keep on conducting research in order to contribute to the adaptation of the system to meet other local specific needs.

## 3. Results and Discussion

Table 1 summarizes results of the questionnaire evaluating Mobil Diab by patients. Each question was evaluated based on a 10-point-scale from 1 to 10 point with high scores reflecting positive feedback from the user. Three metrics were considered for evaluation: usability and design, efficiency and therapy satisfaction, and acceptance and appreciations. Score regarding usability and design was the average score for questions 1, 2, 3, 4, and 5 and resulted in a mean value of 7 points out of ten. This score is the average from all patients of the intervention group. Score for efficiency and therapy satisfaction was the average score for questions 6 and 7 and resulted in a mean value of 7.43 points out of ten. At last, score related to acceptance and appreciations was averaged from questions 11 and 12 and resulted in a mean value of 8.65 points out of ten. These results reflect an overall positive feedback of the patients about the system.

Table 2 represents the evaluation from the medical staff involved in the study. These include four medical doctors, two nurses, and two nutritionists. Score regarding usability and design was the average score for questions 1 and 2 and resulted in a mean value of 7.56 points out of ten. This score is the average from all medical care personnel. Score for efficiency and therapy satisfaction was the average score for questions 3, 4, 5, 6, 7, and 8 and resulted in a mean value of 7 points out of ten. Acceptance and appreciations score was averaged from questions 12 and 13 and resulted in a mean value of 8.75 points out of ten. These results reflect an overall positive feedback of the patients about the system.

The randomization of all the 40 patients in control and intervention group was successful. Both groups are statistically comparable in terms of relevant diabetes therapy.

Table 3 shows the impact of using Mobil Diab on the clinical results. Two metrics were used to assess the improvement in the quality of metabolic control: the mean amplitude of glycemic excursions (characterized from the mean BG and its standard deviation) and the mean HbA1c of each group. We should notice that the mean amplitude of glycemic excursions is the measure of diabetic instability, or a characteristic of blood glucose behavior and the HbA1c is the standard measure of average glycemic control predicting diabetes complications in type 1 and type2.

The approximate mapping between HbA1c values given in percentage (%) and mean BG (estimated average glucose) measurements was calculated using the following equation [17]:

(1) $$\begin{array}{c} \text{HbA1c}\;\left[\%\right]=\frac{\left(\text{mean}\;\text{BG}\left[\text{mg}/\text{dL}\right]+86\right)}{33.3}. \end{array}$$

Positive results have been noticed at the end of the trial, this is particularly evident in a significant improvement in the mean HbA1c values for the intervention group. The mean HbA1c for that group was 8.67% before the start of the trial and could be reduced to 6.89% at the end. This value of 1.78% in the improvement of HbA1c indicates that the risk of diabetes complications has been reduced or postponed, thus leading to cost savings.

Moreover, an important improvement could be noticed in the mean amplitude of glycemic excursions. This is shown in Table 3 by an important reduction of the glucose variability (characterized by the standard deviation of the mean BG). The mean BG standard deviation was 48.6 mg/mL for the control group whereas; this could be reduced to 33 mg/dL for the intervention group at the end of the trial.

Patients found the system as a helpful tool, since it helped them increase their motivation for regular glycemic control, motivated them to fix some goals or targets values for blood glucose control for the future, and helped them to control their meal since they could access easily the database containing the amount of carbohydrate for each meal and portion they were taking. Medical care personnel listed such benefits as follows: the system allowed them to coach several patients at the same time and at a distance, and modernization of health care system allowed them to get rich and useful data through a secure system. The amelioration in the clinical results is due to these above listed benefits provided by the system.

However, some disadvantages such as costs for internet connection and time needed for getting used to the application were the preoccupation of both categories of the users. Wishes from both categories of the subjects included to the trial (medical staff and patients) for the amelioration of the system were collected and included the following:

- Extend the drug list and meal database in the application to include all local foods, and applied drugs, since some of them were missing
- More sensitizing activities and training sessions about the system to patients and medical staff
- Include the support of the glucose measuring device kit in the model
- Create a framework for sport activities for patients

## 4. Conclusions

An IT-enabled diabetes management with fully integrated provider-patient system has been presented in this paper. Results from the trial conducted in two eastern cities of the Democratic Republic of Congo have proved how the system Mobil Diab is suited not only for developed countries but also for communities traditionally underserved, those in remote or rural areas with few health services and staff. This population can access diabetes care using Mobil Diab, because the system overcomes distance and time barriers between health-care providers and patients.

The use of Mobil Diab showed improvement of clinical outcomes of the patients from the intervention group involved in the conducted trial. This has been demonstrated from the amelioration of both the HbA1c (from 8.67% to 6.89%) and the mean amplitude of glycemic excursions which is characterized from both the mean blood glucose and its standard deviation. The decrease of the blood glucose fluctuations is demonstrated from results of the mean blood glucose standard deviation from the intervention group compared to the control group at the end of the study (33.0 mg/mL instead of 48.6 mg/mL). This proves how the use of the system could help patients stabilize better their glucose values.

Positive evaluations of the system from patients and medical staff have been presented based on three metrics: usability and design, efficiency and therapy satisfaction, and acceptance and appreciations. The obtained scores are 7 points and greater out of the 10 maximum points.

The model developed from the conducted pilot in the Democratic Republic of Congo can be extended and applied for other countries in order to enhance awareness on the diabetes management in the continent and to improve the quality of service in the care process.

## Figures and Tables

**Table 1:** Evaluation of usability, efficiency, and acceptance of Mobil Diab by patients.

Evaluation of MobilDiab by patients									
Subjects (patients)	Q1 (points)	Q2 (points)	Q3 (points)	Q4 (points)	Q5 (points)	Q6 (points)	Q7 (points)	Q11 (points)	Q12 (points)
P1	8	9	10	10	9	10	10	10	10
P2	7	5	8	7	5	8	7	8	7
P3	5	3	5	10	5	10	10	10	10
P4	8	8	8	9	6	10	7	10	10
P5	3	4	4	3	6	5	3	5	5
P6	10	10	7	8	10	10	10	10	10
P7	8	7	5	6	9	10	7	10	10
P8	4	4	8	2	4	10	7	10	10
P9	6	5	10	7	8	10	10	10	7
P10	7	8	9	9	8	9	8	9	10
P11	4	5	5	6	3	6	4	5	6
P12	6	7	7	8	10	8	4	9	7
P13	1	5	5	5	5	5	4	6	7
P14	5	6	7	8	8	10	8	10	10
P15	8	9	8	7	8	10	1	10	10
P16	9	10	8	8	8	10	10	10	10
P17	7	8	9	7	6	10	5	10	10
P18	9	10	7	9	8	10	8	9	10
P19	3	5	7	7	8	5	4	5	5
P20	6	6	6	7	8	7	4	8	8
Sum	**124**	**134**	**143**	**143**	**142**	**173**	**131**	**174**	**172**
Average for each question	**6.2**	**6.7**	**7.15**	**7.15**	**7.1**	**8.65**	**6.55**	**8.7**	**8.6**

	Usability and design					Efficiency and therapy satisfaction		Acceptance and appreciations	

Average (max. 10 points)	**7**					**7.43**		**8.65**	

**Table 2:** Evaluation of usability, efficiency, and acceptance of Mobil Diab by the medical staff.

Evaluation of Mobil Diab by the medical staff										
Doctors	Q1 (points)	Q2 (points)	Q3 (points)	Q4 (points)	Q5 (points)	Q6 (points)	Q7 (points)	Q8 (points)	Q12 (points)	Q13 (points)
D1	8	8	7	9	8	8	8	9	9	10
D2	9	7	6	7	7	8	8	5	8	8
D3	7	8	7	8	7	7	8	8	9	7
D4	9	8	2	7	7	8	8	8	7	8
D5	8	8	10	6	5	9	9	5	10	10
D6	6	8	9	9	6	6	7	7	9	9
D7	7	6	3	6	6	7	5	5	8	9
D8	7	7	5	7	6	8	7	8	9	10
Average for each question	**7.63**	**7.5**	**6.12**	**7.37**	**6.5**	**7.62**	**7.5**	**6.87**	**8.63**	**8.87**

	Usability and design		Efficiency and therapy satisfaction						Acceptance and appreciations	

Average (max. 10 points)	**7.56**		**7**						**8.75**	

**Table:** (a) Intervention group: clinical results, changes in the quality of metabolic control after 60 days trial, and mean HbA1C for the intervention group before the beginning of the study which was 8.67%

Patients (sex, age)	Mean BG (mg/dL) day1–day60	Standard deviation (mg/dL)	HbA1C (%) day1–day60
P1 (M, 54)	208.2	91.9	8.8
P2 (F, 38)			
P3 (M, 52)	95.3	1.5	5.4
P4 (M, 58)	76.3	4.0	4.8
P5 (F, 60)	108.8	32.6	5.8
P6 (M, 48)	128.4	44.5	6.4
P7 (M, 55)	303.7	84.4	11.7
P8 (F, 49)			
P9 (M, 48)	130.0	38.4	6.4
P10 (M, 62)	141.4	48.0	6.8
P11 (M, 51)	180.1	15.8	7.9
P12 (M, 56)	134.2	38.0	6.6
P13 (M, 57)			
P14 (M, 43)	111.5	11.1	5.9
P15 (M, 60)	132.7	32.6	6.5
P16 (M, 58)	128.8	30.3	6.4
P17 (M, 50)	157.4	30.0	7.3
P18 (F, 35)	111	10.9	5.9
P19 (M, 70)	108	14.5	5.8
P20 (M, 62)	116.3	31.8	6.0

**53.3** Mean age, St. deviation 10.7	143.5 Mean blood glucose	33.0 Mean BG St. deviation	**6.89** Mean HbA1C at the end

**Table:** (b) Control group: clinical results, changes in the quality of metabolic control after 60 days trial, and mean HbA1C for the control group before the beginning of the study which was 8.59%

Patients (sex and age)	Mean BG (mg/dL) day1–day60	Standard deviation (mg/dL)	HbA1C (%) day1–day60
Pc1 (M, 43)	261.8	44.2	10.4
Pc2 (M, 55)	187.2		8.2
Pc3 (M, 60)	174	48.2	7.8
Pc4 (F, 37)	227.8	33.2	9.4
Pc5 (M, 48)			
Pc6 (M, 67)	175.7	17.7	7.8
Pc7 (F, 40)	159	29.5	7.3
Pc8 (M, 42)	202.9		8.6
Pc9 (M, 57)	299.5	101.4	11.5
Pc10 (F, 53)			
Pc11 (M, 59)			
Pc12 (M, 66)			
Pc13 (M, 54)	163.2	35.1	7.4
Pc14 (M, 45)			
Pc15 (F, 58)	269.4	80.6	10.6
Pc16 (M, 61)	184.2	68.4	8.1
Pc17 (M, 75)	181.3	71.8	8.0
Pc18 (F, 49)			
Pc19 (F, 52)	180.4	12.6	8
Pc20 (F, 46)	160	40	7.3

**53.35** Mean age, St. deviation 9.59	201.9 Mean blood glucose	48.6 Mean BG St. deviation	**8.6** Mean HbA1C at the end

(St. deviation: standard deviation, mean BG: mean blood glucose values).
